# Missouri Valley Veterinary Association

**Published:** 1896-02

**Authors:** S. L. Hunter

**Affiliations:** Secretary, Fort Leavenworth, Kan.


### MISSOURI VALLEY VETERINARY ASSOCIATION.

ABOUT forty members of the profession and friends attended the seventh regular meeting of the Missouri Valley Veterinary Association, at Kansas City, Mo., December 13, 1895, when a most interesting programme was fully carried out. President Stewart called the meeting to order at 7 P.M.

After roll-call and adoption of the minutes of the previous meeting, the papers were read and each one discussed separately. Dr. Harrison's paper was a report of a post-mortem examination of a black jack, and many specimens were shown that indicated the cause of death beyond a doubt; Dr. G. T. Netherton treated "Purpura Hemorrhagica in the Bovine Species;" Dr. L. M. Klutts, "Fractures Complex, with Miracles," also a case of protrusion of the bowels as a result of an injury; Dr. Charles Saunders, "Care of Mares in Foal;" Dr. L. R. Brady, "Canine Distemper;" Dr. S. Stewart, "Meat Inspection." All the papers were excellent, and showed great care in preparation, and were fully discussed, bringing out many valuable points.

In all respects it was an excellent meeting and all present were greatly benefited. It was fully demonstrated that no one can afford to miss any meeting of the Missouri Valley Veterinary Association, and the interest is constantly increasing. The next regular meeting will be held in Kansas City in February, 1896.

S. L. HUNTER, V.S., Secretary,
Fort Leavenworth, Kan.

